# Supplementary material for: Causal Relationship between Aging and Anorexia Nervosa: A White-Matter-Microstructure-Mediated Mendelian Randomization Analysis
Source: Biomedicines. 2024 Aug 16;12(8):1874. doi: 10.3390/biomedicines12081874 (PMC11351342; doi:10.3390/biomedicines12081874)
Supplement: Supplementary file 1 [file biomedicines-12-01874-s001.zip › Supplementary Figure/Figure S4/Singe SNP plot of MR analysis for TL to ubm-b-1612.pdf]

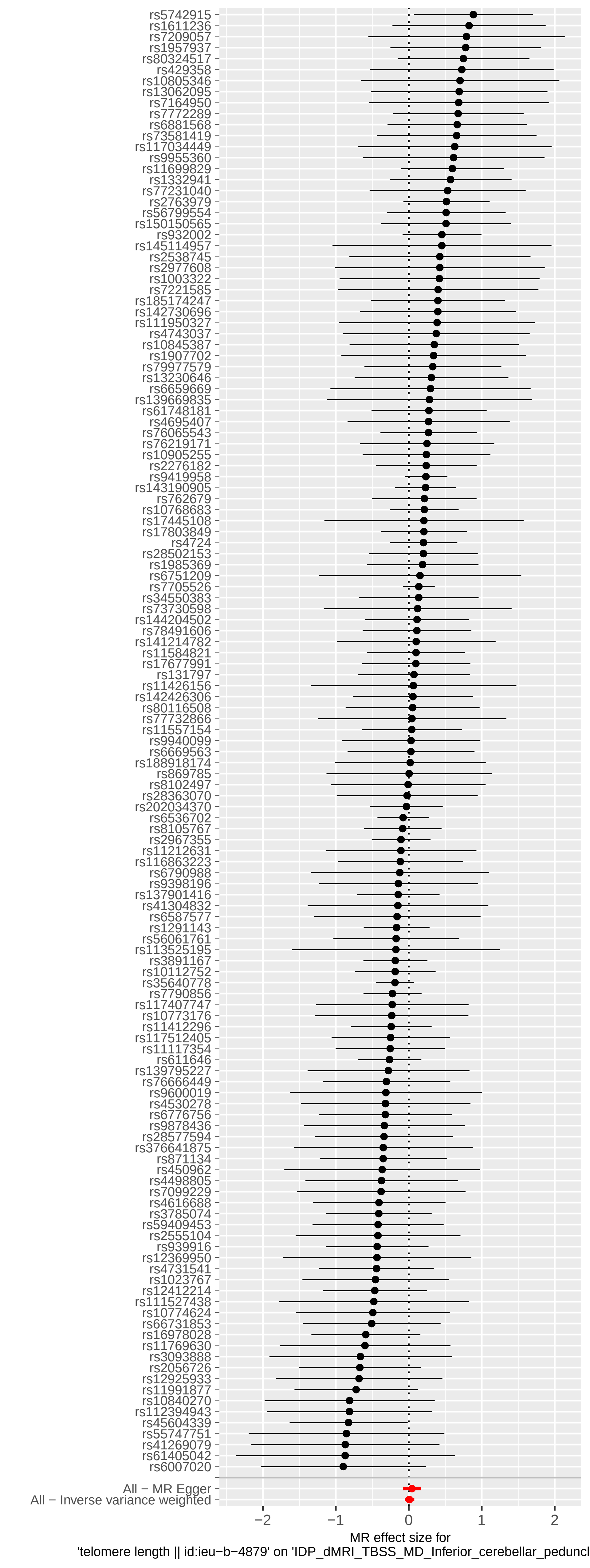
rs5742915
rs1611236
rs7209057
rs1957937
rs80324517
rs429358
rs10805346
rs13062095
rs7164950
rs7772289
rs6881568
rs73581419
rs117034449
rs9955360
rs11699829
rs1332941
rs77231040
rs2763979
rs56799554
rs150150565
rs932002
rs145114957
rs2538745
rs2977608
rs1003322
rs7221585
rs185174247
rs142730696
rs111950327
rs4743037
rs10845387
rs1907702
rs79977579
rs13230646
rs6659669
rs139669835
rs61748181
rs4695407
rs76065543
rs76219171
rs10905255
rs2276182
rs9419958
rs143190905
rs762679
rs10768683
rs17445108
rs17803849
rs4724
rs28502153
rs1985369
rs6751209
rs7705526
rs34550383
rs73730598
rs144204502
rs78491606
rs141214782
rs11584821
rs17677991
rs131797
rs11426156
rs142426306
rs80116508
rs77732866
rs11557154
rs9940099
rs6669563
rs188918174
rs869785
rs8102497
rs28363070
rs202034370
rs6536702
rs8105767
rs2967355
rs11212631
rs116863223
rs6790988
rs9398196
rs137901416
rs41304832
rs6587577
rs1291143
rs56061761
rs113525195
rs3891167
rs10112752
rs35640778
rs7790856
rs117407747
rs10773176
rs11412296
rs117512405
rs11117354
rs611646
rs139795227
rs76666449
rs9600019
rs4530278
rs6776756
rs9878436
rs28577594
rs376641875
rs871134
rs450962
rs4498805
rs7099229
rs4616688
rs3785074
rs59409453
rs2555104
rs939916
rs12369950
rs4731541
rs1023767
rs12412214
rs111527438
rs10774624
rs66731853
rs16978028
rs11769630
rs3093888
rs2056726
rs12925933
rs11991877
rs10840270
rs112394943
rs45604339
rs55747751
rs41269079
rs61405042
rs6007020
All − MR Egger
All − Inverse variance weighted
−2
−1
0
1
2
MR effect size for
'telomere length || id:ieu-b-4879' on 'IDP_dMRI_TBSS_MD_Inferior_cerebellar_peduncl
